# Supplementary material for: Modeling glioblastoma heterogeneity as a dynamic network of cell states
Source: Mol Syst Biol. 2021 Sep 16;17(9):e10105. doi: 10.15252/msb.202010105 (PMC8444284; doi:10.15252/msb.202010105)
Supplement: Supplementary file 6 — Source Data for Figure 5 [file MSB-17-e10105-s004.zip › Figure5A_sourcedata/GSEA_3071/nosigdb_stateI.GseaPreranked.1622795145664/VANGRONINGEN_MES.html]

Details for gene set VANGRONINGEN\_MES[GSEA]

|  || Dataset | state13071 |
| Phenotype | NoPhenotypeAvailable |
| Upregulated in class | na\_pos |
| GeneSet | VANGRONINGEN\_MES |
| Enrichment Score (ES) | 0.50811434 |
| Normalized Enrichment Score (NES) | 3.5267534 |
| Nominal p-value | 0.0 |
| FDR q-value | 0.0 |
| FWER p-Value | 0.0 |
Table: GSEA Results Summary

  

Fig 1: Enrichment plot: VANGRONINGEN\_MES      
 Profile of the Running ES Score & Positions of GeneSet Members on the Rank Ordered List

  

| PROBE | GENE SYMBOL | GENE\_TITLE | RANK IN GENE LIST | RANK METRIC SCORE | RUNNING ES | CORE ENRICHMENT || 1 | MGP |  |  | 1 | 2.652 | 0.0145 | Yes |
| 2 | COL3A1 |  |  | 3 | 2.646 | 0.0291 | Yes |
| 3 | COL1A1 |  |  | 6 | 2.008 | 0.0397 | Yes |
| 4 | COL6A1 |  |  | 10 | 1.797 | 0.0488 | Yes |
| 5 | FN1 |  |  | 11 | 1.769 | 0.0587 | Yes |
| 6 | COL6A3 |  |  | 13 | 1.717 | 0.0681 | Yes |
| 7 | COL5A1 |  |  | 14 | 1.706 | 0.0776 | Yes |
| 8 | BGN |  |  | 24 | 1.568 | 0.0836 | Yes |
| 9 | COL4A1 |  |  | 28 | 1.524 | 0.0912 | Yes |
| 10 | CFH |  |  | 29 | 1.523 | 0.0998 | Yes |
| 11 | POSTN |  |  | 32 | 1.494 | 0.1075 | Yes |
| 12 | COL11A1 |  |  | 37 | 1.468 | 0.1145 | Yes |
| 13 | STK38L |  |  | 39 | 1.441 | 0.1223 | Yes |
| 14 | TIMP1 |  |  | 47 | 1.381 | 0.1278 | Yes |
| 15 | COL5A2 |  |  | 48 | 1.373 | 0.1355 | Yes |
| 16 | TPBG |  |  | 51 | 1.346 | 0.1425 | Yes |
| 17 | CDH11 |  |  | 59 | 1.296 | 0.1476 | Yes |
| 18 | APOE |  |  | 60 | 1.294 | 0.1548 | Yes |
| 19 | PLXDC2 |  |  | 61 | 1.265 | 0.1619 | Yes |
| 20 | TFPI |  |  | 64 | 1.241 | 0.1682 | Yes |
| 21 | COL6A2 |  |  | 67 | 1.237 | 0.1745 | Yes |
| 22 | OLFML2A |  |  | 68 | 1.227 | 0.1814 | Yes |
| 23 | ITGA10 |  |  | 70 | 1.217 | 0.1879 | Yes |
| 24 | COL4A2 |  |  | 73 | 1.203 | 0.1940 | Yes |
| 25 | PPIC |  |  | 77 | 1.195 | 0.1998 | Yes |
| 26 | NRP1 |  |  | 81 | 1.180 | 0.2055 | Yes |
| 27 | ID3 |  |  | 86 | 1.152 | 0.2107 | Yes |
| 28 | F2RL2 |  |  | 87 | 1.137 | 0.2171 | Yes |
| 29 | FLRT2 |  |  | 89 | 1.131 | 0.2231 | Yes |
| 30 | COL12A1 |  |  | 91 | 1.117 | 0.2291 | Yes |
| 31 | PHLDA3 |  |  | 93 | 1.116 | 0.2350 | Yes |
| 32 | ZFP36L1 |  |  | 97 | 1.096 | 0.2402 | Yes |
| 33 | SOSTDC1 |  |  | 99 | 1.094 | 0.2460 | Yes |
| 34 | DDR2 |  |  | 104 | 1.075 | 0.2508 | Yes |
| 35 | F2R |  |  | 105 | 1.067 | 0.2568 | Yes |
| 36 | SPARC |  |  | 106 | 1.067 | 0.2628 | Yes |
| 37 | NQO1 |  |  | 113 | 1.045 | 0.2668 | Yes |
| 38 | S1PR3 |  |  | 114 | 1.043 | 0.2726 | Yes |
| 39 | ITGAV |  |  | 117 | 1.029 | 0.2778 | Yes |
| 40 | SVIL |  |  | 120 | 1.014 | 0.2828 | Yes |
| 41 | EMP1 |  |  | 122 | 1.012 | 0.2882 | Yes |
| 42 | OLFML3 |  |  | 125 | 0.971 | 0.2930 | Yes |
| 43 | CTSB |  |  | 129 | 0.964 | 0.2975 | Yes |
| 44 | ITPRIPL2 |  |  | 130 | 0.962 | 0.3029 | Yes |
| 45 | GPX8 |  |  | 133 | 0.959 | 0.3076 | Yes |
| 46 | FAM129A |  |  | 135 | 0.953 | 0.3127 | Yes |
| 47 | KDELR3 |  |  | 136 | 0.951 | 0.3180 | Yes |
| 48 | FBN1 |  |  | 138 | 0.944 | 0.3230 | Yes |
| 49 | NPC2 |  |  | 139 | 0.943 | 0.3283 | Yes |
| 50 | SNAI2 |  |  | 142 | 0.940 | 0.3329 | Yes |
| 51 | MGST1 |  |  | 150 | 0.915 | 0.3359 | Yes |
| 52 | OSTC |  |  | 151 | 0.915 | 0.3410 | Yes |
| 53 | SERPINH1 |  |  | 157 | 0.902 | 0.3445 | Yes |
| 54 | VCL |  |  | 162 | 0.891 | 0.3483 | Yes |
| 55 | PCDH18 |  |  | 171 | 0.870 | 0.3507 | Yes |
| 56 | FAM114A1 |  |  | 174 | 0.864 | 0.3549 | Yes |
| 57 | EGFR |  |  | 175 | 0.864 | 0.3597 | Yes |
| 58 | IGFBP5 |  |  | 198 | 0.833 | 0.3576 | Yes |
| 59 | SLC38A2 |  |  | 200 | 0.826 | 0.3619 | Yes |
| 60 | CALD1 |  |  | 201 | 0.824 | 0.3665 | Yes |
| 61 | EMILIN1 |  |  | 207 | 0.812 | 0.3695 | Yes |
| 62 | RRBP1 |  |  | 208 | 0.810 | 0.3741 | Yes |
| 63 | OLFML2B |  |  | 233 | 0.763 | 0.3710 | Yes |
| 64 | AEBP1 |  |  | 236 | 0.761 | 0.3746 | Yes |
| 65 | UGDH |  |  | 238 | 0.759 | 0.3785 | Yes |
| 66 | B2M |  |  | 241 | 0.744 | 0.3821 | Yes |
| 67 | LMNA |  |  | 247 | 0.739 | 0.3847 | Yes |
| 68 | TM4SF1 |  |  | 249 | 0.735 | 0.3885 | Yes |
| 69 | SCPEP1 |  |  | 259 | 0.727 | 0.3898 | Yes |
| 70 | GSN |  |  | 265 | 0.720 | 0.3923 | Yes |
| 71 | IFITM3 |  |  | 268 | 0.714 | 0.3957 | Yes |
| 72 | ATP2B1 |  |  | 270 | 0.713 | 0.3994 | Yes |
| 73 | MMP2 |  |  | 284 | 0.697 | 0.3993 | Yes |
| 74 | RBMS1 |  |  | 285 | 0.697 | 0.4032 | Yes |
| 75 | P4HA1 |  |  | 291 | 0.688 | 0.4055 | Yes |
| 76 | KANK2 |  |  | 299 | 0.673 | 0.4071 | Yes |
| 77 | DSE |  |  | 312 | 0.659 | 0.4071 | Yes |
| 78 | TPM2 |  |  | 320 | 0.655 | 0.4086 | Yes |
| 79 | GRN |  |  | 321 | 0.655 | 0.4122 | Yes |
| 80 | MICAL2 |  |  | 322 | 0.651 | 0.4159 | Yes |
| 81 | LRP10 |  |  | 329 | 0.646 | 0.4177 | Yes |
| 82 | ATP2B4 |  |  | 330 | 0.644 | 0.4213 | Yes |
| 83 | HSPA5 |  |  | 337 | 0.638 | 0.4230 | Yes |
| 84 | PALLD |  |  | 342 | 0.636 | 0.4253 | Yes |
| 85 | WLS |  |  | 360 | 0.625 | 0.4236 | Yes |
| 86 | SHC1 |  |  | 361 | 0.624 | 0.4271 | Yes |
| 87 | SERPINE2 |  |  | 364 | 0.624 | 0.4300 | Yes |
| 88 | BTN3A2 |  |  | 366 | 0.624 | 0.4331 | Yes |
| 89 | ITGB1 |  |  | 371 | 0.615 | 0.4354 | Yes |
| 90 | FLNA |  |  | 373 | 0.614 | 0.4385 | Yes |
| 91 | FILIP1L |  |  | 387 | 0.602 | 0.4379 | Yes |
| 92 | ANXA5 |  |  | 392 | 0.597 | 0.4400 | Yes |
| 93 | SEMA3C |  |  | 402 | 0.586 | 0.4405 | Yes |
| 94 | CKAP4 |  |  | 405 | 0.582 | 0.4431 | Yes |
| 95 | SQSTM1 |  |  | 412 | 0.578 | 0.4445 | Yes |
| 96 | LAMB1 |  |  | 415 | 0.577 | 0.4471 | Yes |
| 97 | FKBP14 |  |  | 418 | 0.575 | 0.4497 | Yes |
| 98 | TPM1 |  |  | 419 | 0.575 | 0.4529 | Yes |
| 99 | GDF15 |  |  | 424 | 0.574 | 0.4549 | Yes |
| 100 | LMAN1 |  |  | 438 | 0.559 | 0.4540 | Yes |
| 101 | ELF1 |  |  | 439 | 0.558 | 0.4572 | Yes |
| 102 | PPIB |  |  | 442 | 0.555 | 0.4597 | Yes |
| 103 | NOTCH2 |  |  | 446 | 0.551 | 0.4618 | Yes |
| 104 | PDIA3 |  |  | 467 | 0.537 | 0.4587 | Yes |
| 105 | CTSO |  |  | 472 | 0.532 | 0.4604 | Yes |
| 106 | EDNRA |  |  | 479 | 0.529 | 0.4615 | Yes |
| 107 | TMBIM4 |  |  | 492 | 0.520 | 0.4607 | Yes |
| 108 | PRDX4 |  |  | 494 | 0.519 | 0.4633 | Yes |
| 109 | RIN2 |  |  | 499 | 0.513 | 0.4650 | Yes |
| 110 | TMEM263 |  |  | 502 | 0.509 | 0.4672 | Yes |
| 111 | FSTL1 |  |  | 505 | 0.504 | 0.4694 | Yes |
| 112 | TSC22D3 |  |  | 513 | 0.500 | 0.4701 | Yes |
| 113 | DNAJC3 |  |  | 533 | 0.489 | 0.4669 | Yes |
| 114 | PDLIM1 |  |  | 539 | 0.487 | 0.4681 | Yes |
| 115 | HNMT |  |  | 540 | 0.487 | 0.4709 | Yes |
| 116 | LAPTM4A |  |  | 553 | 0.478 | 0.4698 | Yes |
| 117 | HTRA1 |  |  | 555 | 0.477 | 0.4722 | Yes |
| 118 | TJP1 |  |  | 557 | 0.477 | 0.4746 | Yes |
| 119 | TM9SF2 |  |  | 569 | 0.470 | 0.4738 | Yes |
| 120 | TMEM50A |  |  | 577 | 0.466 | 0.4743 | Yes |
| 121 | LOXL2 |  |  | 589 | 0.459 | 0.4734 | Yes |
| 122 | ARMCX2 |  |  | 598 | 0.454 | 0.4735 | Yes |
| 123 | PLS3 |  |  | 612 | 0.446 | 0.4720 | Yes |
| 124 | THBS1 |  |  | 613 | 0.446 | 0.4745 | Yes |
| 125 | CD63 |  |  | 618 | 0.444 | 0.4757 | Yes |
| 126 | WIPI1 |  |  | 620 | 0.443 | 0.4779 | Yes |
| 127 | FIBIN |  |  | 630 | 0.441 | 0.4776 | Yes |
| 128 | PCSK5 |  |  | 636 | 0.439 | 0.4785 | Yes |
| 129 | LAMC1 |  |  | 640 | 0.439 | 0.4801 | Yes |
| 130 | KLF6 |  |  | 647 | 0.433 | 0.4806 | Yes |
| 131 | EGR1 |  |  | 652 | 0.432 | 0.4818 | Yes |
| 132 | YAP1 |  |  | 657 | 0.430 | 0.4830 | Yes |
| 133 | CYBRD1 |  |  | 672 | 0.422 | 0.4810 | Yes |
| 134 | ID1 |  |  | 682 | 0.420 | 0.4806 | Yes |
| 135 | DNAJC10 |  |  | 684 | 0.419 | 0.4827 | Yes |
| 136 | MAGT1 |  |  | 686 | 0.418 | 0.4847 | Yes |
| 137 | PXDN |  |  | 690 | 0.416 | 0.4861 | Yes |
| 138 | EPS8 |  |  | 691 | 0.414 | 0.4884 | Yes |
| 139 | SLC39A14 |  |  | 695 | 0.414 | 0.4898 | Yes |
| 140 | MYADM |  |  | 697 | 0.413 | 0.4918 | Yes |
| 141 | ATP10D |  |  | 698 | 0.412 | 0.4941 | Yes |
| 142 | CREB3L2 |  |  | 699 | 0.412 | 0.4964 | Yes |
| 143 | LIPA |  |  | 700 | 0.412 | 0.4987 | Yes |
| 144 | CMTM3 |  |  | 713 | 0.407 | 0.4973 | Yes |
| 145 | ANXA6 |  |  | 719 | 0.405 | 0.4980 | Yes |
| 146 | GJA1 |  |  | 727 | 0.403 | 0.4981 | Yes |
| 147 | KDELR2 |  |  | 737 | 0.401 | 0.4976 | Yes |
| 148 | KLF4 |  |  | 742 | 0.400 | 0.4986 | Yes |
| 149 | ARPC1B |  |  | 743 | 0.400 | 0.5009 | Yes |
| 150 | TWSG1 |  |  | 747 | 0.398 | 0.5022 | Yes |
| 151 | TMED9 |  |  | 759 | 0.394 | 0.5010 | Yes |
| 152 | PON2 |  |  | 770 | 0.392 | 0.5001 | Yes |
| 153 | COPA |  |  | 771 | 0.392 | 0.5023 | Yes |
| 154 | TRAM1 |  |  | 780 | 0.389 | 0.5020 | Yes |
| 155 | ATP6V0E1 |  |  | 788 | 0.386 | 0.5020 | Yes |
| 156 | SSR1 |  |  | 795 | 0.384 | 0.5023 | Yes |
| 157 | FUCA2 |  |  | 797 | 0.383 | 0.5041 | Yes |
| 158 | HSP90B1 |  |  | 798 | 0.382 | 0.5063 | Yes |
| 159 | CLIC4 |  |  | 800 | 0.382 | 0.5081 | Yes |
| 160 | ARL1 |  |  | 813 | 0.377 | 0.5065 | No |
| 161 | EXTL2 |  |  | 818 | 0.375 | 0.5074 | No |
| 162 | SURF4 |  |  | 830 | 0.371 | 0.5061 | No |
| 163 | IFITM2 |  |  | 836 | 0.370 | 0.5066 | No |
| 164 | MRC2 |  |  | 863 | 0.361 | 0.5006 | No |
| 165 | BNC2 |  |  | 864 | 0.361 | 0.5026 | No |
| 166 | ADAMTS5 |  |  | 876 | 0.357 | 0.5012 | No |
| 167 | PAPSS2 |  |  | 898 | 0.351 | 0.4967 | No |
| 168 | ARHGEF40 |  |  | 899 | 0.351 | 0.4987 | No |
| 169 | LIFR |  |  | 904 | 0.350 | 0.4994 | No |
| 170 | SLC30A7 |  |  | 908 | 0.349 | 0.5005 | No |
| 171 | CSRP1 |  |  | 921 | 0.345 | 0.4987 | No |
| 172 | SLC35F5 |  |  | 926 | 0.343 | 0.4994 | No |
| 173 | SPCS3 |  |  | 927 | 0.343 | 0.5013 | No |
| 174 | TCF7L2 |  |  | 932 | 0.343 | 0.5020 | No |
| 175 | PXDC1 |  |  | 952 | 0.339 | 0.4980 | No |
| 176 | LUZP1 |  |  | 958 | 0.337 | 0.4984 | No |
| 177 | CTDSP2 |  |  | 959 | 0.337 | 0.5003 | No |
| 178 | HYOU1 |  |  | 961 | 0.336 | 0.5018 | No |
| 179 | ATP8B2 |  |  | 978 | 0.333 | 0.4988 | No |
| 180 | ELK3 |  |  | 998 | 0.328 | 0.4947 | No |
| 181 | L3HYPDH |  |  | 1008 | 0.326 | 0.4938 | No |
| 182 | GALNT10 |  |  | 1047 | 0.319 | 0.4839 | No |
| 183 | IGFBP6 |  |  | 1048 | 0.318 | 0.4856 | No |
| 184 | MYL12A |  |  | 1053 | 0.316 | 0.4862 | No |
| 185 | DDOST |  |  | 1055 | 0.316 | 0.4876 | No |
| 186 | LIX1L |  |  | 1057 | 0.316 | 0.4891 | No |
| 187 | IFI16 |  |  | 1062 | 0.315 | 0.4896 | No |
| 188 | MYDGF |  |  | 1067 | 0.313 | 0.4902 | No |
| 189 | PPT1 |  |  | 1071 | 0.312 | 0.4910 | No |
| 190 | VIM |  |  | 1105 | 0.305 | 0.4825 | No |
| 191 | NPTN |  |  | 1127 | 0.302 | 0.4777 | No |
| 192 | PLAGL1 |  |  | 1132 | 0.301 | 0.4782 | No |
| 193 | CD164 |  |  | 1133 | 0.301 | 0.4799 | No |
| 194 | STAT1 |  |  | 1136 | 0.301 | 0.4809 | No |
| 195 | PRDM6 |  |  | 1144 | 0.299 | 0.4804 | No |
| 196 | JAK1 |  |  | 1145 | 0.299 | 0.4821 | No |
| 197 | NR3C1 |  |  | 1162 | 0.297 | 0.4789 | No |
| 198 | TMEM87B |  |  | 1163 | 0.297 | 0.4805 | No |
| 199 | NEK7 |  |  | 1176 | 0.293 | 0.4785 | No |
| 200 | ZCCHC24 |  |  | 1187 | 0.290 | 0.4770 | No |
| 201 | ARL4A |  |  | 1195 | 0.289 | 0.4765 | No |
| 202 | HIBADH |  |  | 1197 | 0.289 | 0.4778 | No |
| 203 | TXNDC12 |  |  | 1201 | 0.288 | 0.4785 | No |
| 204 | IL6ST |  |  | 1215 | 0.285 | 0.4760 | No |
| 205 | REST |  |  | 1225 | 0.285 | 0.4749 | No |
| 206 | RNFT1 |  |  | 1236 | 0.282 | 0.4734 | No |
| 207 | RCN1 |  |  | 1250 | 0.277 | 0.4709 | No |
| 208 | LEPROT |  |  | 1278 | 0.272 | 0.4641 | No |
| 209 | PLOD2 |  |  | 1304 | 0.268 | 0.4579 | No |
| 210 | CPED1 |  |  | 1309 | 0.267 | 0.4582 | No |
| 211 | CYR61 |  |  | 1318 | 0.265 | 0.4572 | No |
| 212 | SLC16A4 |  |  | 1325 | 0.264 | 0.4568 | No |
| 213 | RIT1 |  |  | 1337 | 0.262 | 0.4549 | No |
| 214 | ACTN1 |  |  | 1364 | 0.258 | 0.4483 | No |
| 215 | FZD7 |  |  | 1374 | 0.256 | 0.4470 | No |
| 216 | PLPP1 |  |  | 1395 | 0.252 | 0.4422 | No |
| 217 | CBLB |  |  | 1398 | 0.252 | 0.4430 | No |
| 218 | RAP1B |  |  | 1400 | 0.252 | 0.4441 | No |
| 219 | TGFBR2 |  |  | 1410 | 0.251 | 0.4427 | No |
| 220 | CD44 |  |  | 1430 | -0.251 | 0.4383 | No |
| 221 | ENAH |  |  | 1599 | -0.267 | 0.3880 | No |
| 222 | ERRFI1 |  |  | 1640 | -0.273 | 0.3772 | No |
| 223 | POLR2L |  |  | 1742 | -0.285 | 0.3476 | No |
| 224 | ABRACL |  |  | 1753 | -0.287 | 0.3462 | No |
| 225 | KCTD12 |  |  | 1756 | -0.288 | 0.3472 | No |
| 226 | A2M |  |  | 1808 | -0.295 | 0.3331 | No |
| 227 | ADGRE5 |  |  | 1912 | -0.307 | 0.3030 | No |
| 228 | PTBP1 |  |  | 1934 | -0.311 | 0.2983 | No |
| 229 | CTSC |  |  | 1975 | -0.315 | 0.2877 | No |
| 230 | SUCLG2 |  |  | 1988 | -0.316 | 0.2858 | No |
| 231 | ADAM9 |  |  | 2063 | -0.328 | 0.2648 | No |
| 232 | PYGL |  |  | 2071 | -0.329 | 0.2645 | No |
| 233 | ETS1 |  |  | 2086 | -0.332 | 0.2620 | No |
| 234 | FBN2 |  |  | 2221 | -0.357 | 0.2227 | No |
| 235 | TRIL |  |  | 2250 | -0.363 | 0.2161 | No |
| 236 | HOMER1 |  |  | 2313 | -0.374 | 0.1991 | No |
| 237 | TSC22D2 |  |  | 2328 | -0.379 | 0.1969 | No |
| 238 | ELAVL1 |  |  | 2588 | -0.436 | 0.1195 | No |
| 239 | SSBP4 |  |  | 2596 | -0.438 | 0.1198 | No |
| 240 | EXT1 |  |  | 2615 | -0.443 | 0.1167 | No |
| 241 | JAM3 |  |  | 2654 | -0.455 | 0.1075 | No |
| 242 | PLK2 |  |  | 2720 | -0.476 | 0.0902 | No |
| 243 | APP |  |  | 2770 | -0.495 | 0.0778 | No |
| 244 | TRAM2 |  |  | 2810 | -0.504 | 0.0686 | No |
| 245 | SHROOM3 |  |  | 2905 | -0.545 | 0.0427 | No |
| 246 | SPRY4 |  |  | 2916 | -0.552 | 0.0427 | No |
| 247 | PRCP |  |  | 2919 | -0.553 | 0.0452 | No |
| 248 | GAS1 |  |  | 3009 | -0.607 | 0.0211 | No |
| 249 | ROBO1 |  |  | 3016 | -0.610 | 0.0227 | No |
| 250 | ITPR1 |  |  | 3029 | -0.619 | 0.0225 | No |
| 251 | PLEKHA2 |  |  | 3049 | -0.634 | 0.0202 | No |
| 252 | ATP1B1 |  |  | 3093 | -0.669 | 0.0107 | No |
| 253 | PTGFRN |  |  | 3111 | -0.685 | 0.0093 | No |
| 254 | NFIC |  |  | 3133 | -0.707 | 0.0067 | No |
| 255 | SPRY1 |  |  | 3163 | -0.746 | 0.0020 | No |
| 256 | DUSP6 |  |  | 3227 | -0.816 | -0.0129 | No |
| 257 | LITAF |  |  | 3247 | -0.843 | -0.0140 | No |
| 258 | SOX9 |  |  | 3271 | -0.883 | -0.0162 | No |
| 259 | SEL1L3 |  |  | 3279 | -0.892 | -0.0133 | No |
| 260 | MEST |  |  | 3307 | -0.940 | -0.0164 | No |
| 261 | PTPRG |  |  | 3308 | -0.945 | -0.0111 | No |
| 262 | PROM1 |  |  | 3347 | -1.035 | -0.0170 | No |
| 263 | TNC |  |  | 3350 | -1.043 | -0.0118 | No |
| 264 | TMEFF2 |  |  | 3410 | -1.267 | -0.0229 | No |
| 265 | DLX1 |  |  | 3455 | -1.622 | -0.0274 | No |
| 266 | DLX2 |  |  | 3474 | -1.814 | -0.0227 | No |
| 267 | PTN |  |  | 3476 | -1.843 | -0.0127 | No |
| 268 | SCRG1 |  |  | 3480 | -1.876 | -0.0032 | No |
| 269 | NFIA |  |  | 3492 | -2.214 | 0.0059 | No |
Table: GSEA details [plain text format]

  

Fig 2: VANGRONINGEN\_MES: Random ES distribution      
 Gene set null distribution of ES for **VANGRONINGEN\_MES**
